# ‘Don’t let it hold you back’ — The experience of transition to adulthood in young people with primary ciliary dyskinesia: An interpretative phenomenological analysis

**DOI:** 10.1177/13591053231223912

**Published:** 2024-01-28

**Authors:** Rhys Dore, Isabella E Nizza, Hannah M Mitchison, Celine Lewis

**Affiliations:** 1 Genetics & Genomic Medicine, UCL Great Ormond Street Institute of Child Health, London, UK; 2 Department of Psychological Sciences, Birkbeck University of London, London, UK; 3 Population, Policy and Practice, UCL Great Ormond Street Institute of Child Health, London, UK; 4 North Thames Genomic Laboratory Hub, Great Ormond Street Hospital for Children NHS Foundation Trust, London, UK

**Keywords:** adolescence, chronic illness, experience, health care systems, illness perception, interpretative phenomenological analysis (IPA), qualitative methods, respiratory problems, stigmatisation

## Abstract

Primary ciliary dyskinesia (PCD) is a rare, chronic genetic condition with variable features arising from motile cilia dysfunction, including recurrent respiratory infections, sinonasal disease, reduced hearing, infertility and situs inversus. The aim of the study was to understand the experiences of young people with PCD as they transition into adulthood and adult healthcare services. An interpretative phenomenological analytical method was applied. Semi-structured interviews were conducted with three participants aged 18–24 years. Four interconnected group experiential themes were identified: (1) reconceptualising a stigmatised identity, (2) sharing the journey to independence, (3) entering adulthood with newfound autonomy, (4) anticipating an uncertain future. Overall, we found that transition for young people with PCD presents as a complex period marked by identity-formation, creating systems of support and becoming an autonomous adult. Facilitation of personalised and integrated approaches to care should be prioritised. Our findings are important to help health professionals provide appropriate, anticipatory support.

## Introduction

Primary ciliary dyskinesia (PCD) is a rare genetic disease caused by structural changes in the motile airway cilia, resulting in their immotility or dysmotility and markedly reducing mucociliary clearance (Lucas et al., 2014). This leads to chronic upper and lower respiratory infection and progressive severe lung damage (Halbeisen et al., 2018). PCD is heterogeneous both in its genetic aetiology and its phenotypic manifestation (Horani and Ferkol, 2021). The cardinal features include unexplained neonatal respiratory distress, laterality defects, daily non-seasonal nasal congestion and year-round wet cough (Leigh et al., 2016). Bronchiectasis can present in infancy and is almost invariable by adulthood, predominantly affecting the lower and middle lobes (Lucas et al., 2014). Symptomatology can include sinonasal disease, otological disease, hearing problems and infertility. Treatment is typically via regular chest physiotherapy, nebulisers and inhalers, and prolonged antibiotic courses for infection (Paff et al., 2021). Associated symptoms can be treated by using hearing aids for hearing loss and access to fertility clinics if required. There are currently no curative or disease-modifying treatments. Life expectancy does not seem to be significantly reduced although there is a paucity of evidence regarding this, and severe untreated lung disease may increase mortality risk. Lung lobectomy can be considered for severe, localised bronchiectasis and bilateral lung transplantation is an option in cases of end-stage respiratory failure (Lucas et al., 2014).

Understanding the experience of PCD is vital for identifying areas for support and improvement. Multiple qualitative studies provide insights by investigating experiences such as diagnosis, treatment and daily life (Behan et al., 2016, 2017; Dell et al., 2014; Lucas et al., 2015). However, these often evaluate the experiences of adults with PCD or the experience of parenting a child with PCD – neither of which may be applicable to young adults. Key insights from adults include the importance of symptomatology and psychosocial experiences in the perception of stigmatisation (McManus et al., 2006; Whalley and McManus, 2006). In terms of children and young people, parental accounts have often been prioritised. One thematic analysis of interview data from people with PCD or their parents gave credence to the diagnostic odyssey which is often present within rare diseases and the relief and burden associated with diagnosis (Behan et al., 2016). Further reports evaluating parental experiences also noted heightened anxiety regarding diagnosis during the covid-19 pandemic (Driessens et al., 2022). Similar studies identified barriers to treatment such as limited belief in efficacy, time burden and wanting a ‘normal’ life (Taelman et al., 2014a, 2014b).

Whilst the established research is enlightening, multiple studies focus on reports from adults, and it is evident that the literature qualitatively demonstrates a variety of experiences of living with PCD. Adults are by default at a starkly different life stage to young people, who are newly navigating the complexity of independence. In the UK, formal transition to adult healthcare services is completed by age 16–18 years, increasingly via the ‘Ready, Steady, Go’ preparatory framework (Nagra et al., 2015). Previous IPA (interpretative phenomenological analysis) research has focussed on children before formal transition (Schofield and Horobin, 2014). However, unlike younger children, young adults are at a key turning point where they must take and maintain responsibility whilst entering general adulthood and this comes with changes to education, relationships, location and even identity. Whilst questionnaires have evaluated the presence of psychological symptomatology in adolescents with PCD, there is limited qualitative data for this group (Valero-Moreno et al., 2020). Research regarding transition has often considered diseases that cannot faithfully recapitulate the experience of having PCD, such as cystic fibrosis which has different pathogenesis, treatment options and prognosis (Brumfield and Lansbury, 2004). Transition-related research regarding PCD has instead focussed on service development (Prentice et al., 2018; Schmalstieg and Omran, 2021). Additionally, the young people of today will have uniquely undergone transition during the covid-19 pandemic. Whilst the COVID-PCD consortium collected survey data regarding experiences during this time, there are limited rich qualitative enquiries into the effects on young people (Pedersen et al., 2021b). Early results highlighted that people living in the UK, especially children, were most likely to report covid-19 infection, but that most participants reported minimal symptoms (Pedersen et al., 2023).

Adolescence and young adulthood are critical stages for all young people for their identity formation and establishment of their place within their social milieu. For those with chronic illnesses, the typical stages of development are intertwined with their health experience. Health and illness perceptions can shape the way in which people understand themselves, their lives and their positions within society. For young people affected by PCD, examining these experiences can form the basis of understanding the origin of these perceptions. The Common Sense Model (CSM) considers the involvement of illness perceptions in reason-making from appraisals of health threat representations, coping strategies and resulting outcomes (Leventhal et al., 2016). Further insight can be gained by determining to what extent such models of illness cognition either match or contrast the lived experiences.

Therefore, the aim of this research is to explore the lived experiences of young people with PCD who have undergone both transition into adult healthcare services and transition into general adulthood, an area currently understudied. This can provide insights into not only healthcare, but also into how PCD interacts with other life experiences such as education, relationships, identity and the covid-19 pandemic. We hope that this greater understanding will help to identify areas of need to provide further appropriate support for young people with PCD.

## Methods

### Methodology

IPA was chosen as it allows investigating in depth the lived experiences of participants via rich personal accounts, uncovering, via a double-hermeneutic, different layers of meaning attached to these experiences (Smith, 2018; Smith et al., 2022). The idiographic focus of IPA allows depth of enquiry for the small sample sizes of rare disease research. Additionally, established markers of high-quality IPA research ensure rigorous application of the methodology (Nizza et al., 2021).

### Ethics

This project was reviewed and approved by the UCL Ethics Committee under Project Number 22113/001. Informed consent for publication was provided via a RedCap form and verbally.

### Recruitment

Participants were recruited via the charity PCD Support UK (2022). There were two rounds of advertising on the charity’s website, social media pages (Facebook, Twitter, Instagram) and via their mailing list. Whilst the reach of PCD Support UK is broad, only those based within the UK were invited to participate. Participants were provided with a £10 Amazon voucher for participation. Potential participants who completed an expression of interest form were sent a participant information sheet and formal consent was collected for those who agreed to participate via RedCap – additionally, consent was verbally re-confirmed before starting each interview.

### Inclusion criteria

Participants were eligible if aged 18–25 years, diagnosed with PCD during childhood (<18 years of age), and based in the UK. This age range was chosen as these young people would have most recently undergone transition into both adult healthcare services and adulthood. Participants were excluded if they were currently receiving or had previously received treatment for a diagnosed mental health condition considering the risk of distress during the interview.

### Interviews

Semi-structured interviews were undertaken using video-conferencing software. The topic guide was reviewed and piloted with the chair of PCD Support UK. Topics included life with PCD, identity, transition to adult healthcare services, transition to adulthood, relationships, experiences through the covid-19 pandemic and the future. See Supplemental Materials for the complete interview guide. During the interview, any unexpected relevant topics raised by participants were also explored. Great Ormond Street Hospital Psychological Services were available for signposting as necessary.

### Analytic process

Audio recordings were transcribed verbatim following each interview to ensure recollection of context (Rodham et al., 2014). Each transcript analysis was undertaken before moving on to the next as suggested by IPA best practice (Smith and Nizza, 2022). This involved re-readings to ensure close attention to the data with at least two rounds of exploratory note formation. Experiential statements were formed which described and interpreted the experiences. These were printed and manually collated to identify clusters via an iterative process. Clustered experiential statements were named to form personal experiential themes (PETs) with subthemes. These were entered into an Excel spreadsheet and transcript quotes were identified to ensure that PETs reliably represented the raw data. Individual PETs and subthemes were compared and clustered within Excel to create overarching group experiential themes (GETs). The iterative process of modification continued throughout creation of the final manuscript.

### Participants

Three participants were included, aged 18–24 years. Two participants identified as female and one as male. Two participants were of white British ethnicity and one was of Asian/Asian British ethnicity. Participants had been diagnosed with PCD between the ages of 1 and 11 years. Pseudonyms have been used to maintain anonymity. Given the rarity of PCD and the community being small, details about participants are intentionally generic, to reduce their risk of being identifiable.

## Results

Six people expressed interest and three (50%) agreed and consented to take part. Interviews lasted approximately 80 minutes, excluding introduction and debrief, and they were audio recorded. Four GETs were identified in the data. All themes were relevant to each participant:

1. Reconceptualising a stigmatised identity
2. Sharing the journey to independence
3. Entering adulthood with newfound autonomy
4. Anticipating an uncertain future

### Theme 1: Reconceptualising a stigmatised identity

Assimilation of a PCD identity was variable. Whilst life with PCD was marred by stigmatisation and efforts for concealment, it could be positively reconceptualised.

Participants reported significant consideration in divulging their diagnosis. Beth emphasised this by stating ‘*my lung condition, my business*’. The importance of control over this private information was echoed by George:

*[PCD is] something that’s quite personal to me [. . .] I don’t really know why that is. I think because [. . .] a lot of people around me know quite a bit about [healthcare] and stuff like that so I feel like, I keep, kind of keep it a bit more to myself now [George]*

For George, PCD took on the form of a ‘secret’ that could only be shared with trusted friends. Those with increased awareness of PCD within his peer group who had not been invited to know this ‘secret’ were seen as unintentionally intrusive. As a core part of their ‘personal’ identity, PCD revealed a hidden part of themselves. Participants’ avoidance of describing PCD by referring to it as ‘*asthma*’ or a ‘*lung condition*’ maybe was not simply due to the complexity of describing a rare disease, but was perhaps representative of preferring to present themselves with a label commonly recognised and accepted within society rather than what could be perceived as the more fearful unknown of a rarely understood condition.

Nonetheless, concealment was not static as participants became more open over time with trusted persons. Amira highlighted the balance between remaining ‘*low key*’ whilst learning to share her identity:

*I like to keep everything low key [. . .] if I want people to know they’ll know, but if I don’t want them to know something they won’t [. . .] as I’ve grown up, I’ve become a lot more open about my condition as well which is a good thing in some cases. [Amira]*

Whilst some aspects of PCD could be hidden, participants highlighted that symptomatology could attract negative attention:

*I think some people [. . .] the bullies or just nasty people [. . .] see it maybe as disgusting. And I’ve heard that, I’ve been called that, so I know that. [Beth]*

Her use of ‘*maybe*’ initially suggests uncertainty in her assumptions about the thoughts of others. Yet, she then quickly confirms her worries by extrapolating specific experiences to the general context of bullying – demonstrating internalisation of external stigma. This internalisation was also expressed by George:

*. . . as I’m older I’ve always worried about like how it does sound a bit disgusting and like the fact that you’ve got nothing in your lungs to like help clear out all the crap and the crap collects at the bottom of your lungs, it basically becomes like a breeding ground for bacteria to the point that I’m colonised and stuff like that. It sounds, I think it sounds a bit dirty, but actually, in retrospect [. . .] is it? Like you know everyone’s got bacteria and stuff like that. My body’s just not as good at getting rid of it and actually I’d say I’m quite a clean hygienic person.* [George]

This passage was marked by temporal ambiguity and contradictions. George engaged in a close dialogue between two identities – his identity as a healthcare worker versus his identity as a person with PCD. He mentioned the past, ‘*I’ve always worried*’, in reference to his concerns before entering healthcare and the concerns of those around him considering PCD as ‘*disgusting*’. This changed to the present tense with a brief description of the underlying pathophysiology by focussing on ‘*the fact. . .*’. Regardless, the vocabulary used shows non-medical personal interpretations of the ‘*crap*’ in the lungs and thinking ‘*it sounds a bit dirty*’. This contrasted with the medically sanctioned term ‘*colonised*’ which may otherwise be negatively loaded for those outside of healthcare – likening the lungs to a site of infestation. Despite his medical rationalisation of bacterial presence within the lungs, he continued to close his comment by stating that he was a ‘*clean hygienic person*’. Thus, showing that despite his knowledge as a healthcare worker, he still struggled with internalised stigma that allowed his ‘*dirty*’ lungs to encapsulate his sense of self. This dual identity of patient and healthcare worker has made George’s struggle to assimilate PCD even more difficult.

Amira too demonstrated an ongoing concern regarding how she was perceived:

*I would get picked on quite a lot because I guess when you’re a kid you don’t know how to clear your nose either. And I’d always be coughing and stuff [Amira]*

Rather than directing blame towards those who mistreated her, she blamed herself and her inability to clear her nose – displaying an expectation and an acceptance of being bullied. Whilst bullying for Amira and Beth was most prominent during earlier schooling, George explained how the covid-19 pandemic presented a new challenge of misconceptions about his coughing representing covid-19 infection:

*I think during [covid-19] I thought about it a bit more and thought about the whole, you know I don’t blame anyone, but it’s like that whole hidden disability thing and stuff like that again, like people don’t realise what’s going on. [George]*

Whilst the PCD diagnosis may have been ‘*hidden*’, the symptomatology was contrastingly overt resulting in increasing external stigmatisation. Once again, no ‘*blame*’ was attributed and there was a begrudged acceptance that this was expected in light of having a ‘*hidden disability*’. This contrasted with the preference for concealment of his diagnosis, demonstrating the fluidity of these concerns. Whilst participants may have perceived more common conditions as garnering less external scrutiny, covid-19 was instead deemed to have more negative perceptions than PCD.

The experience of growing up with PCD undoubtedly shaped participants’ identities, yet not necessarily in a similar fashion. In response to internalised stigmatisation, George aimed to distance himself from PCD:

*I don’t want it to be an issue, I don’t want it to be a big part of my life. I’ve spent most of my life trying to live like a normal person [George]*

He focussed on presenting a façade of what he perceived to be ‘*normal*’ and refuted PCD having a place within this perceived normality. This was further encapsulated by his suggestion that ‘*it shouldn’t define you, it’s just part of you*’. This emphasised that the reach of PCD should be compartmentalised and restricted, albeit still a part of his identity. It was similar to any other personal trait, even if not a ‘*normal*’ one. This recognition of PCD as a key trait is somewhat paralleled by Beth who stated:

*It also becomes part of you and part of who you are and you’ll end up loving it as a [. . .] quirk that you have. [Beth]*

However, the key contrast here was that Beth believed that PCD could be integrated successfully into her identity by deeming it an essential and positive aspect of herself. PCD remained a ‘*part*’ of her but rather than discussions surrounding normality, she framed PCD as a ‘*quirk*’ – an interesting and unique but not necessarily deleterious feature. Amira too preferred to focus on the ‘*positive*’ aspects:

*I feel like if I didn’t have PCD I wouldn’t be as independent and maybe as positive. Yeah. So I feel like it has shaped me as a person but it’s not been in a bad way. [Amira]*

All participants attributed positive changes such as ‘*independence*’ and ‘*organisation*’ to PCD. Interestingly, the focus on PCD not shaping Amira in a ‘*bad*’ way, rather than the affirmation of it being good, highlighted her expectation that outsiders anticipated PCD to be a negative force – a concept that she raised and rebutted. Thus, assimilation of PCD into one’s identity did not mandate focus upon negative or illness-related aspects. Instead, all participants reconceptualised the concept of disease identity. They experienced and understood the restrictiveness of living their lives through perceived or actualised stigma and sought to purposely present their identities as more than this.

Overall, all participants experienced stigma associated with their condition throughout their childhood, adolescence and present. This was complicated by the rarity of their condition resulting in a lack of understanding amongst their peers, which became more problematic during the covid-19 pandemic. Despite varied struggles, all participants reached a point where they recognised that PCD was part of their identity. Their reflections upon this assimilation demonstrated that such acceptance exists on a complex spectrum from begrudged admittance to open-armed affirmation. Regardless, their ability to reconceptualise PCD and its prominence illustrated a stark contrast to their experience of stigma.

### Theme 2: Sharing the journey to independence

Living with a rare disease can be lonely considering the lack of those who can truly empathise with the experience. However, the participants recounted many ways in which these experiences were shared with family members, friends and others in their support network.

Early in the diagnostic period, Beth described feeling ‘*in the dark*’ regarding PCD and her hospitalisation. However, this loneliness did not refer to her singly, but also to her parents whom she described as not having had enough support. Parents acted as researchers, educators, protectors and cheerleaders. They developed expertise in PCD that was recognised by their children and shared with them. Beth described her mother:

*I ask her for any advice about medical conditions and she kind of warns me what to say, what not to say [. . .] she encourages me to get active and things [. . .] she’s always on my side with these things like bad doctors [. . .] she’ll shout at them for me. That’s the kind of parent you want. [Beth]*

Her mother was on the same ‘*side*’ as her. This painted the picture of being at war against PCD, oppressive systems and inflexible or outright ‘*bad*’ healthcare – and finding solace and safety in family. Her reference to ‘*bad doctors*’ suggested the existence of ‘good doctors’ that conversely find themselves within her positive support network.

Ongoing support was also provided by friends who were seen as accepting. Beth discussed how this search for acceptance resulted in ‘*becom[ing] closer with your friends and your family*’. However, Amira highlighted the strain that the covid-19 pandemic placed on friendships:

*[my friends] used to go out and stuff and obviously I was still shielding, so then it’s like hmm that’s like awkward. . . [Amira]*

She showed her internal conflict in admitting the ‘*awkward*’ feeling of being left out despite a recognition that she could not join due to shielding. The risk related to PCD acted as a barrier to an otherwise fulfilling friendship – removing the familiar faces that she expected to join her as she entered adulthood.

All participants had family members or friends who were affected by PCD and described the shared understanding and camaraderie that they found in these unique relationships. For example, George stated:

*I’m kind of glad that we both had it in a way. Rather, obviously, we didn’t, but, you know, if that makes sense. It is something to go through, someone to talk to things about. [George]*

Whilst he felt guilty in his happiness about a family member having PCD, he could not deny the sense of togetherness and solidarity that this brought. Support also felt particularly effective from friends with PCD as described by Amira:

*. . . with my non PCD friends, like I can tell them stuff but they’re not fully going to get it, but like obviously with the other ones, they understand and you don’t really have to say much. [Amira]*

Not needing ‘*to say much*’ to be understood was representative of shared experiences where empathy is granted without the need for laborious explanations. Effective support required understanding.

This concept of shared experiences and their importance continued into the transition phase:

*I think we [were] almost waiting for that day where we transition. Almost scared me a bit because I knew that it was meant to be not very good. [George]*

There was anxiety and anticipation in the ‘*waiting*’. Transition became a fearful yet inevitable event. Interestingly, George used the words ‘*where we transition*’, implying that the transition process was not solely related to him, but for his family as a unit. However, the fear he described is personal. As the participants matured, they became increasingly aware of the importance of seeking information about their care and took an increasingly central role. Beth had prepared a range of questions to lessen the unknown:

*I had my last children’s visit [. . .] I made sure to ask questions [. . .] that made me feel more kind of confident about it. [Beth]*

With this inquisitiveness, all participants became ‘expert’ patients and increasingly recognised deficiencies within health professionals’ knowledge. However, Amira raised that knowledge extends past just medical expertise:

*. . . your whole care got passed over to someone who didn’t know anything about you because it’s just one clinic appointment but then they learnt a lot and got to know us all the same like within the first 6 months [Amira]*

Knowledge was a bidirectional process. ‘*Whole care*’ carried the weight of meaning for both clinical healthcare and individual personhood. There is a person behind the disease and ‘*just one clinic appointment*’ seemed too rudimentary to recognise this. Whilst she initially considered transition ‘*scary*’, there was relief in realising that it was ‘*not that bad*’ – despite ‘*bad*’ having been the expectation. This change of heart appeared to be from the recognition that a relationship must be forged with your healthcare provider and that this was successful.

Overall, participants did not undertake the journey to adulthood alone. They received support from family members, trusted friends and ‘vetted’ health professionals. Additionally, they all either had or sought companionship from others affected by PCD. Whilst this reduced the perceived sense of loneliness, it is also worth noting that unaffected family members often became infused in the experience of living with PCD. This allowed effective modelling and teaching so that participants could take the next steps in seizing responsibility.

### Theme 3: Entering adulthood with newfound autonomy

On entering adulthood, participants became increasingly responsible for their care with variable progression in their autonomy over different aspects of their lives. With this newfound power, they made decisions that complemented their planned trajectories.

Despite descriptions of early rebellion, participants became more mature in adulthood. Amira said:

*I’m more responsible for my care [. . .] when I was younger my mum would sometimes say she’d exercise me and stuff. Not that I’d actually listen when I was younger. Now I know for myself how important it is, so I do it without anyone telling me. [Amira]*

She highlighted the change in mentality upon adulthood – undertaking PCD treatment changed from being externally forced into something for which she had internal motivation.

Having a chronic disease meant that the participants became adept at treatment at a young age – a proxy for adult-like responsibilities. Beth described being ‘*treated [. . .] more maturely*’ and considered more adult than her peers.

George described how having already taken some responsibility for his own care lessened his perceived burden:

*I guess if someone hadn’t had to ever deal with all this stuff then suddenly had to start dealing with it, [it] probably would be quite daunting but I think you just [. . .] get used to it. [George]*

Treatment just became ‘*part of [his] routine*’. Despite seeming objectively ‘*daunting*’, this did not concern him because he was already accustomed to his treatment regime. All participants concurred regarding the routinisation of treatment – however, this confidence did not necessarily translate to confidence within a healthcare setting, as Amira had experienced during the covid-19 pandemic:

*. . . you had to go to clinic alone [. . .] which wasn’t something I used to do. But now it’s fine [. . .] it doesn’t bother me. But at first, I was like that’s scary [Amira]*

Amira initially felt out of her depth in clinic, which was like her experience of adult wards which felt ‘*different*’ and ‘*intense*’ after becoming accustomed to paediatric settings. Notably, she still felt seen as the ‘*baby of the family*’. This emotion was shared by Beth who remarked that she was ‘*technically still a child*’ at the point of taking responsibility within clinic. Transition to adulthood and transition to adult healthcare services were not the same. Becoming an adult within healthcare could be marked by becoming an imposter – a ‘faux adult’ who was still coming to terms with general adulthood, let alone adulthood within healthcare.

This was especially noteworthy when considering that chronic conditions have impacts that extend past healthcare provision. George described difficulty in losing support whilst balancing other responsibilities of general adulthood:

*I came into university having this extra support and then it was just kind of all dropped which is hard when you’re already trying to adjust to university life [George]*

He referred to the transition as a ‘*kick in the teeth*’, suggesting that autonomy in managing health does not necessarily mean autonomy in all aspects of life, nor that support was no longer required.

For Beth, there remained a need to prove herself when away from her parents:

*. . . [travelling abroad with school] made me become more confident about myself and about travelling and also about being able to look after myself. I think my parents also became more confident about it as well and kind of felt more secure about me maybe going off doing my own thing [Beth]*

Despite becoming an expert in her own treatment, other activities possibly had a higher threshold for parental trust in view of established anxiety about managing with PCD.

Nonetheless, as participants became more acquainted with their autonomy, they became increasingly pragmatic about the interaction between PCD and their blossoming adult lives. For example, Beth allowed some sacrifices in her physiotherapy to maintain the lifestyle she wanted and reported it was ‘*as good as it’s going to get*’.

With increasing responsibility came increasing recognition of when their preferences were not acknowledged. George described wanting to continue his university course despite external concerns regarding covid-19:

*It didn’t seem fair and equal and that actually, I feel like it was a risk that I felt was actually okay and I was prepared to take. [George]*

These factors became noticeable when life plans, such as careers, did not align with external expectations – covid-19 placed a spotlight on this. Beth and George matured into ownership of themselves and their ‘*risk*’. Beth also faced this struggle within her career choice:

*… it took a while to convince [my parents] especially my mum [. . .] because obviously infection spreads quickly and easily around children [. . .] But I stuck with it and I argued my case and they just accepted that I’m not going to change my mind. [Beth]*

Whilst their personal susceptibilities to covid-19 and other respiratory infections were recognised by George and Beth, the importance of choice took precedence. They refused to allow PCD to restrict dreams and aspirations. Additionally, there was a struggle against their perceived vulnerability with cases being ‘*argued*’ for. Seemingly in contrast, Amira considered the covid-19 pandemic as a time for reflection about careers:

*I thought about [university] and then I just decided [. . .] without thinking too much [. . .] I told my mum “oh I’m not going to go” because of these reasons, like uni’s really long and then like obviously with the pandemic and I feel like it’s not going to be the best teaching [. . .] I feel like it’ll be a waste. [Amira]*

Whilst Amira overtly suggested avoiding overthinking, she provided multiple reasons for not attending university rather than being solely due to perceived vulnerability; she ultimately was ‘*grateful*’ for the pandemic. Whilst Beth and George reinforced their plans, Amira changed hers. The key constant between them was the element of choice.

Overall, participants developed increasing levels of autonomy within their lives. Whilst they were particularly adept in routine treatment, this was not necessarily matched in more complex healthcare interactions or elsewhere in life. This variable progression blurred the period during which their self-perception moved from child to adult and complicated their identity during this period in which they somewhat identified as both. Interestingly, all participants marked their childhood by the inability to undertake their own treatment. However, entry into adulthood was a more complex, multi-faceted and broader definition compared to mastering treatment alone. Nonetheless, as adults they all benefitted by using their autonomy to make pragmatic decisions that aligned with their expected futures.

### Theme 4: Anticipating an uncertain future

The rarity and variability of PCD produces uncertainty regarding future morbidity. Participants recognised and feared a future of potentially worsening health and unrealised expectations.

Becoming key decision-makers in their lives forced the participants to look further into the future. George described the unsettling enlightenment of learning about a particularly restricted person with PCD:

*I met a particular woman [. . .] at the PCD group who [. . .] struggled with [. . .] activities of daily living [. . .] it scared me a bit so that was probably the big turning point for me, and I always remember those discussions [. . .] I think going forward into adult life that’s the thing that I do think about sometimes now [. . .]. What’s the prognosis? Like what am I going to be like in 20 to 30 years because I, at the moment, I’m, you know, pretty fit and healthy [George]*

His potential future ‘unhealthy’ self was at odds with his current self-perception as ‘*fit and healthy*’ and he feared a metamorphosis. His interjection, ‘*at the moment*’, demonstrated his recognition that this metamorphosis may indeed unwillingly occur. Modelling the future based upon other individuals also occurred with Amira who found it both ‘*eye-opening*’ and ‘*shocking*’ to learn about the severe deterioration of another young person with PCD. These foreboding insights acted as a theoretical negative reinforcement of not ‘adhering’ to treatment. This fear of the future started early for those who recalled diagnosis. Beth said:

*I don’t really want to make a huge plan because everything can change. I know that from being diagnosed, everything can change in a month. [Beth]*

Her perceived good health and freedom from disease were unexpectedly deemed fallacious – creating uncertainty around the rest of life. She avoided making a ‘*huge plan*’, suggesting that there was still some yearning to plan the future – as also supported by her chosen career plans. When considering the future, Amira stated:

*I don’t know. I’ve not really thought about it, but I’ve thought about it [Amira]*

There was a contradiction regarding thinking about the future, with resulting hesitancy against being taken adrift upon future aspirations that may or may not be realised. The recognition of uncertainty extended past education and careers, as George explained:

*In terms of wider life, I hope to potentially have kids one day. Not thinking about that now. But yeah, maybe in the next decade which sounds scary. . . [George]*

He raised the ‘*potentially big*’ issue of infertility and contradicted this with ‘*not thinking*’ whilst being aware of his hopeful plans. Ripples of anxiety could be sensed about an untoward outcome. It became increasingly apparent that this was not an avoidance of planning the future, but rather a fear to admit one’s plans considering their possible impermanence. Whilst participants certainly viewed themselves as adults at this stage, the concept of there being a ‘*wider life*’ demonstrated the many stages that are present during adulthood.

Beth too discussed the possible future difficulties broadly related to romantic relationships:

*I know there’ll be guys that might not be okay with [PCD] and I understand that and I’m chill with it [. . .] it is what it is at the end of the day [Beth]*

Instead of feeling anxious about the future, she anticipated that potential partners may not accept a partner with PCD. Interestingly, rather than feeling offended, she appeared relatively content. She accepted that whilst the future cannot always be predicted, there are indeed some aspects that appear unchanging such as her diagnosis or the opinions of others.

Amira echoed this:

*. . . you might plan something but then you might be poorly or you don’t know, like you don’t know what’s around the corner so you appreciate everything a lot more than the average person I’d say. [Amira]*

Repeated emphasis was given to not knowing. The hopeful plans of an anticipated future remained a mystery, whereas the expectation of becoming ‘*poorly*’ was constant – whether it happened now or later, it would happen – imploring a degree of fatalism. Despite this, Amira extracted the silver lining of living in the moment. The value of the present could truly be appreciated when all may be lost. George too focussed upon the need to value the now and aimed to:

*… maintain good health as much as I can, keep up the things that I enjoy doing, and I guess maintain happiness [George]*

He linked perceived health with the ability to conduct enjoyable activities. Considering his preferred hobby was sports, it was evident that the ability to maintain perceived good health was subjective. This may contrast with somebody who is already more restricted – where the ability to undertake activities of daily living becomes the threshold for health. His repetition of ‘*maintain*’ denoted that his focus was not necessarily on improving life but instead on avoiding deterioration. Whilst he already seemed happy, it appeared that anticipation of a future with PCD reduced the expectations of the future becoming even better than the present.

Upon recognising themselves as adults, participants also foresaw the potential complexities and limitations of their futures. This anticipation was in stark contrast to their drive to make decisions that enhanced the likelihood of achieving their life goals. Despite overcoming adversity and restriction, they were faced with further barriers. However, these barriers may come at any time or not at all. Whilst this may be true for all people, the early experiences of ill health and exposure to others with worsening health created an especially poignant conundrum. Participants were forced to embrace a cognitive dissonance in both fighting for their dreams, whilst also anticipating limitations. Whilst avoidance was used as a coping mechanism, the participants found themselves advancing through life and were thus increasingly compelled to relish the present.

## Discussion

This is the first study using IPA to examine the experiences of young people with PCD approaching adulthood. It provides a unique insight into the process of transition both in healthcare and in general life. Participants demonstrated concealment and stigmatisation related to their condition. Despite contrasting incorporation of PCD into identity, they often reconceptualised it as positive. Support was sought for and found in family, friends and others with shared experiences. Transition itself involved recognising their responsibilities and implementing personal choices. Whilst their trajectories differed, they shared a concerted yearning to succeed against a perceived uncertain future.

### Application to research and theory

Whilst participants discussed concealment changing upon ageing, the awareness of stigma appeared to be established early and to continue throughout adulthood as suggested in previous research (Schofield and Horobin, 2014; Whalley and McManus, 2006).

The reconceptualisation of the illness role is particularly interesting. Participants demonstrated variable integration of PCD into their identity. PCD was more compartmentalised by George, and more incorporated into identity by Beth and Amira. Despite this, all referred to personal positive traits that they attributed to having PCD. Finding personal strength in disease has been demonstrated in other chronic conditions (Horky et al., 2017; Kristjansdottir et al., 2018). Optimism can be protective against deleterious physical and mental health – these studies are often in older populations with heart disease or cancer, but evidence has been found in adolescents with cystic fibrosis (Schiavon et al., 2016). In contrast to Weinstein’s ‘unrealistic optimism’ regarding underestimations of future ill health, this optimism instead embraces the historical and potential ill health whilst reframing the outcomes (Weinstein, 1980). The rarity, complexity and relative isolation of their condition allowed participants to develop pride in overcoming adversity and developing a unique skillset. Whilst participants did not all accept being defined by PCD, they willingly accepted being defined by the positive personal outcomes of living with a chronic disease. This concept is akin to post-traumatic growth of which three key domains were also found in our participants: relating to others, personal strength and an appreciation for life (Tedeschi and Calhoun, 1996). Many traditional models incorporate a concept of ‘consequences’ from ill health which suggests predominant negativity. The modern interpretation of the Common Sense Model (CSM) explains how people self-regulate their illness management and considers emotions but typically refers to fear and anxiety and suggests controlling such emotions via coping (Leventhal et al., 2016). Our findings indicate the need to consider a fuller breadth of feelings such as determination, resilience and courage when interpreting health behaviours in young people.

Participants demonstrated a need for personalised and coordinated support services and an augmented knowledge base across the healthcare community. This is replicated within many rare disease communities (Smits et al., 2022). Relief from the diagnostic odyssey does not necessarily bring the coordinated support that is anticipated with a diagnosis (Morris et al., 2022). This is emphasised by the mistrust of the medical profession reported in previous PCD research (Whalley and McManus, 2006). Whilst patients may have the ‘decisional autonomy’ to optimise their health whilst maintaining personal endeavours, having the ‘executive autonomy’ to do so in practice requires knowledge of complex systems (Naik et al., 2009; Valero, 2019). Establishing knowledge within all involved stakeholders improves understanding of needs. Participants recognised this early and quickly developed the skills to educate themselves, their healthcare providers and their community. Upon entering adulthood, they refused to be left ‘*in the dark*’ any longer and realised that they must become their own advocates. In accordance with the self-determination theory applied to health behaviour, participants demonstrated both technical competence and the importance of relatedness by forming supportive networks early in life (Deci and Ryan, 1985; Patrick and Williams, 2012). The vital change was the movement of treatment motivation from parent to child, with participants mastering their therapies long before adulthood.

At medical transition, participants had already moved past the theoretical neurocognitive stage of adolescent risk-taking versus adherence (Steinberg, 2008). They instead faced the complex role of becoming self-governed adults in pursuit of their respective chosen lives in which the possible risks to their health were at odds with the risk of being unfulfilled. ‘Risk’ itself is a simplification considering that each day of life with PCD was peppered with potential risks or exposures, particularly augmented by the covid-19 pandemic, and thus clinical risk was normalised and integrated into their standard decision-making. According to the CSM, health ‘threats’ can be inappropriately minimised through poor rationalisation, but in this study, they are instead considered as expected and anticipated outcomes of a life well-lived, resulting in acceptance rather than behaviour alteration (Leventhal et al., 2016). This appears to contradict the PCD literature which implies a cautiousness regarding clinical vulnerability (Pedersen et al., 2021a, 2021b). However, it must be reiterated that participants were not reckless, but rather their new-found power and increasing maturity allowed them to focus on thriving and flourishing.

A contrast was drawn between the powerful internal locus of control demonstrated by participants regarding their careers and education, versus the avoidance of considering future health and somewhat fatalistic expectations of deterioration. Thus, revealing that participants were not truly revoking belief in their autonomy. They were instead using a coping mechanism identified within the literature – making ‘functional’ use of fatalistic statements to relieve self-blame in what could be an objectively unpredictable disease (Franklin et al., 2007; Keeley et al., 2009). Their continuing adherence to treatment also suggests this is not necessarily an incoherent state, but a product of uncertainty. Models such as the CSM involve considerations of timelines, yet our participants could not accurately predict the length of theoretically acute phases and they knew that their past experiences may not reflect those of the future (Leventhal et al., 2016). This suggests that the CSM does not quite capture the complexity of a rare disease that can be variable despite treatment, that lacks the widespread prognostic evidence of common diseases, and for which our participants did not have the life experiences of older people to help them develop expectations from their own personal history.

From a temporal perspective, participants rapidly progressed from the adolescent phase of identity formation to somewhat experiencing the final stage of Erikson’s identity development model – being forced to prospectively reflect on what they might miss during their life (Erikson, 1968). This creates the risk of pre-emptive mourning for a life not yet lived. The reconceptualised uncertainty in illness theory is of relevance here (Mishel, 1990). This suggests that contentment with uncertainty could be achieved by considering the uncertainty as a natural part of life, rather than as potentially dangerous. This requires probabilistic and conditional thinking that is supported by peers and healthcare professionals – to allow multiple parallel considerations of a future which may alleviate the sense of loss.

### Application to policy and practice

Participants highlighted the need for an increasingly personalised and integrated approach to their care – in a format that ensured they were fully informed. Considering the early stage of embracing responsibility, it may be worth implementing healthcare transition frameworks such as ‘Ready, Steady, Go’ from a particularly young age akin to the gradual devolvement used by parents (Nagra et al., 2015). This should be linked with repeated assessments regarding the need for social, educational and financial support, considering changing circumstances. The UK is undergoing a transition into developing Integrated Care Systems that attempt to coalesce funding for such support services and it is vital that people with rare diseases are not overlooked in their establishment (Department of Health and Social Care, 2023). Whilst such diseases are individually rare, they are collectively common and therefore collated supportive provisions that help people with PCD may indeed be effective models to also support other people with rare diseases. There are clear expectations that Integrated Care Boards linked to these systems will ensure appropriate funding for transition services (NHS England, 2023). These should ideally incorporate engagement of young people and their families to build trust and learn from lived experiences.

Notably, the UK Government’s Rare Disease Action Plan calls for increased awareness and better coordination (Department of Health & Social Care, 2022). The service specification outlines the need for specialist transition clinics and detailed clinical handovers – supported by positive feedback from patients accessing these (NHS England, 2018; Prentice et al., 2018; Wilkins et al., 2019). The utility of these specialist clinics is that they combine healthcare professionals who understand the relevant rare disease and the likely concerns of those affected – reducing the uneasiness and mistrust that patients and their families may have in seemingly unvetted healthcare professionals.

Finally, transition acts as an invitation into adulthood and young people must be treated accordingly. Their established responsibility and autonomous balancing of perceived risk must be respected. Existential concepts regarding their future and future identity should be addressed at an early stage – providing young people with the ability to discuss their concerns, but also their hopes and expectations. It is vital that we recognise that effective healthcare communication is not a one-way provision of medical advice but entails placing young people on a platform where they feel able to enact change. This must be conducted within the current social and healthcare-related context. One recent example being the need to recognise the unique stigmatising experiences that those with PCD were more likely to experience during the covid-19 pandemic. This is necessary to ensure that supportive provisions are appropriately responsive and reduce the risk of becoming outdated as key concerns change over time. The provision of effective anticipatory change in the healthcare experience will require commitment to ongoing engagement of young people with PCD via established clinical and charitable networks.

### Strengths and limitations

A key strength of this research is that it represents a novel and unique insight into transition for those with PCD. The IPA methodology has provided a comprehensive and rich account of this experience as identified by comparison to formal IPA evaluation methods (Nizza et al., 2021; Smith, 2011). The project has a clear focus utilising rich interview data, each theme has been elaborated with rigorously applied evidence from each participant, and the interpretative narrative attends to both convergence and divergence of participant experience. Recommendations from the chair of PCD Support UK were incorporated and the research was aligned with the needs of the PCD community. Results have either recapitulated or built upon previous research on both PCD and rare or chronic diseases. We have shown that approaching adulthood with PCD is a dynamic experience with implications for identity, interpersonal relationships, independence and perceptions of the future.

IPA benefits from a homogeneous sample and aspects such as gender, ethnicity, socioeconomic status, age of diagnosis and heterogeneity of the condition may influence experience, however specific subsets of these categories were not applied for participant selection in view of the rarity of the condition. Nonetheless, shared GETs were effectively formed suggestive of phenomena that transcend such categories for those affected by PCD. Purposive sampling is standard for IPA, yet this study required participant awareness of PCD Support UK to be involved – thus possibly excluding the experiences of those isolated from community support. Additionally, the interview format may have affected communication. Whilst virtual communication is now commonplace, it may have negatively affected conversational flow – limited internet connectivity meant one participant needed to repeat answers. Also, the length of IPA interviews can be mentally and physically demanding – possibly restricting elaboration in answers given towards the end.

### Future research

In view of the unique and formative experience of transition, it would be beneficial to undertake future qualitative research that either compares participants at differing stages of transition, or that provides repeated insights into the same participants longitudinally during the transition process. This could determine key time points at which intervention could improve experience. Additionally, it would be worth undertaking a broader quantitative evaluation via the established PCD networks to determine whether any disease-related or personal characteristics affect opinion on the transition process (Raidt et al., 2022). This may highlight subgroups that require targeted intervention for further support or qualitative investigation. Furthermore, it is worth considering the opinions and experiences of those with supportive roles for people affected by PCD. This would include undertaking qualitative research with family members, friends and health professionals to further characterise effective support and preventative barriers. Combining this information with integrated care models could ensure co-ordinated support via all services that impact on healthcare and general life.

## Conclusion

Transition is a precarious time for young people with PCD. From an early age, they have undergone a transformative process during which PCD has become variably integrated into their identity. Recognition of previous failed support systems invokes a yearning for knowledge, expertise and the safety net of a supportive community. They continue to learn and embrace their responsibility, becoming autonomous adults who seek control over their chosen yet sometimes uncertain future.

## Supplemental Material

sj-docx-2-hpq-10.1177_13591053231223912 – Supplemental material for ‘Don’t let it hold you back’ — The experience of transition to adulthood in young people with primary ciliary dyskinesia: An interpretative phenomenological analysis Supplemental material, sj-docx-2-hpq-10.1177_13591053231223912 for ‘Don’t let it hold you back’ — The experience of transition to adulthood in young people with primary ciliary dyskinesia: An interpretative phenomenological analysis by Rhys Dore, Isabella E Nizza, Hannah M Mitchison and Celine Lewis in Journal of Health Psychology

sj-docx-3-hpq-10.1177_13591053231223912 – Supplemental material for ‘Don’t let it hold you back’ — The experience of transition to adulthood in young people with primary ciliary dyskinesia: An interpretative phenomenological analysis Supplemental material, sj-docx-3-hpq-10.1177_13591053231223912 for ‘Don’t let it hold you back’ — The experience of transition to adulthood in young people with primary ciliary dyskinesia: An interpretative phenomenological analysis by Rhys Dore, Isabella E Nizza, Hannah M Mitchison and Celine Lewis in Journal of Health Psychology

sj-xlsx-1-hpq-10.1177_13591053231223912 – Supplemental material for ‘Don’t let it hold you back’ — The experience of transition to adulthood in young people with primary ciliary dyskinesia: An interpretative phenomenological analysis Supplemental material, sj-xlsx-1-hpq-10.1177_13591053231223912 for ‘Don’t let it hold you back’ — The experience of transition to adulthood in young people with primary ciliary dyskinesia: An interpretative phenomenological analysis by Rhys Dore, Isabella E Nizza, Hannah M Mitchison and Celine Lewis in Journal of Health Psychology
