# Supplementary material for: ‘Don’t let it hold you back’ — The experience of transition to adulthood in young people with primary ciliary dyskinesia: An interpretative phenomenological analysis
Source: J Health Psychol. 2024 Jan 28;29(9):1029–45. doi: 10.1177/13591053231223912 (PMC11301960; doi:10.1177/13591053231223912)
Supplement: sj-docx-2-hpq-10.1177_13591053231223912 – Supplemental material for ‘Don’t let it hold you back’ — The experience of transition to adulthood in young people with primary ciliary dyskinesia: An interpretative phenomenological analysis [file sj-docx-2-hpq-10.1177_13591053231223912.docx]

**Explanatory Memo**

File: Data with themes (.xlsx)

This file presents the PETs (personal experiential themes) and GETs (group experiential themes) with associated subthemes and quotes.

*Table 1. Description of each sheet*

| George | The PETs, subthemes, and associated quotes for George. |
| --- | --- |
| Beth | The PETs, subthemes, and associated quotes for Beth. |
| Amira | The PETs, subthemes, and associated quotes for Amira. |
| Grouped PET Subthemes | PET subthemes for all participants which have been grouped and given provisional GET titles. |
| GET Iterations | Repeated iterations of GETs and subthemes. |

No syntax file nor log file have been provided. Analysis was not conducted with specific software. Analysis was conducted manually by printing out experiential statements and collating them into groups to form PET subthemes, which were thereafter collated into PETs for each participant. Formation of GETs (and GET subthemes) was conducted by manual collation of all participant PET subthemes and altered via an iterative process during manuscript creation.

The original transcriptions with associated explanatory notes and experiential statements have not been provided as these have large amounts of sensitive and/or personal data that may reveal the identity of participants. Notably, pseudonyms have been used throughout.

File: PCD Interview Guide.docx

This file present the interview guide prepared for conducting the interviews, with associated prompts as bullet points.
