# Supplementary material for: ‘Don’t let it hold you back’ — The experience of transition to adulthood in young people with primary ciliary dyskinesia: An interpretative phenomenological analysis
Source: J Health Psychol. 2024 Jan 28;29(9):1029–45. doi: 10.1177/13591053231223912 (PMC11301960; doi:10.1177/13591053231223912)
Supplement: sj-docx-3-hpq-10.1177_13591053231223912 – Supplemental material for ‘Don’t let it hold you back’ — The experience of transition to adulthood in young people with primary ciliary dyskinesia: An interpretative phenomenological analysis [file sj-docx-3-hpq-10.1177_13591053231223912.docx]

**Interview Guide (with prompts)**

1. Please describe a normal week for you.

- Work/education
- Variability of routine
- Common activities

1. How would you describe life with PCD?

- Management (medical/physio)
- Family, friendships, relationships
- Social life
- Education
- Hobbies
- Other health concerns including mental health

1. How has your life changed since childhood?

- Family/relationships
- Social life
- Hobbies
- Has PCD affected these changes, and if so, how?

1. Tell me about your experience of moving from child to adult healthcare services?

- What kind of support did you receive?
- How did this transition feel?
- What support would you have liked to receive?

1. How would you describe yourself as a person?

- How has this changed over time, if at all?

1. Does PCD affect the way you see yourself?
   - Has PCD had a role in shaping who you are today?
2. How do you think others see you?

- Family and friends
- Health professionals
- What do you wish other people knew about you?

1. What are your hopes and expectations about the future?

- Does PCD affect the way you see your future?
- Do you consider PCD when making decisions about your future?

1. What advice would you give to other young people with PCD who are about to move into adult healthcare services and general adulthood?
2. How has the covid pandemic affected you and the people you care about?
3. To what extent would your answers have been different if there were no pandemic?
4. Is there anything else you would like to say that I haven’t asked about?
